# Perinatal depression screening in community pharmacy: Exploring pharmacists’ roles, training and resource needs using content analysis

**DOI:** 10.1007/s11096-023-01647-0

**Published:** 2023-10-04

**Authors:** Clara Strowel, Camille Raynes-Greenow, Lily Pham, Stephen Carter, Katharine Birkness, Rebekah J. Moles, Claire L. O’Reilly, Timothy F. Chen, Corina Raduescu, Andrea Murphy, David Gardner, Sarira El-Den

**Affiliations:** 1 Sydney School of Public Health, Faculty of Medicine and Health, University of Sydney, Sydney, NSW Australia; 2 The University of Sydney School of Pharmacy, Faculty of Medicine and Health, The University of Sydney, Sydney, NSW Australia; 3 Dalhousie University, Halifax, NS Canada; 4 The University of Sydney Business School, University of Sydney, Sydney, NSW 2050 Australia; 5 College of Pharmacy, Faculty of Health, Dalhousie University, Halifax, NS Canada; 6 Department of Psychiatry, Faculty of Medicine, Dalhousie University, Halifax, NS Canada

**Keywords:** Perinatal depression, Pharmacy, Resources, Screening, Training

## Abstract

**Background:**

Perinatal depression (PND) screening is often recommended in primary care settings, which includes the community pharmacy setting. However, there is limited research exploring pharmacists’ perspectives on their roles in screening for perinatal mental illness.

**Aim:**

This study aimed to explore pharmacists’ views of pharmacists’ roles in PND screening, as well as training and resource needs for PND screening in community pharmacy settings.

**Method:**

A questionnaire including three open-ended questions focusing on pharmacists’ perspectives of their role in PND screening, their training, and resource needs in this area, was disseminated to pharmacists across Australia via professional organisations and social media. Each open-ended question was separately analysed by inductive content analysis. Subcategories were deductively mapped to the Theoretical Framework of Acceptability.

**Results:**

Responses (N = 149) from the first open-ended question about pharmacists’ roles in PND screening resulted in three categories (*PND screening in primary care settings will support the community, community pharmacy environment, and system and policy changes*) and ten subcategories. Responses to question two on training needs (n = 148) were categorised as: *training content, training length, and training delivery* while responses about resource needs (n = 147) fell into three categories: *adapting community pharmacy operating structures, pharmacist-specific resources, and consumer-specific resources*.

**Conclusion:**

While some pharmacists were accepting of a role in PND screening due to pharmacists’ accessibility and positive relationships with consumers, others had concerns regarding whether PND screening was within pharmacists’ scope of practice. Further training and resources are needed to facilitate pharmacists’ roles in PND screening, referral and care.

## Impact statements

- Pharmacist participants were generally accepting of an expanded role in PND screening and care, provided that community pharmacists receive PND-specific training and resources, and referral pathways for community pharmacy are established.
- Pharmacists highlighted a need for further training in PND, specifically around screening processes and communication strategies, and provided suggestions for training content, length and delivery mode.
- Findings of this study can be used to guide the development of pharmacy-specific training and resources around PND screening and care within community pharmacies.

## Introduction

Mental illnesses occurring during pregnancy and up to one year after birth are described as perinatal mental illnesses [1], whereby depressive and anxiety disorders are the most prevalent [2]. In high income countries, between 10 and 15% of women experience perinatal depression (PND) [3]. Most international and Australian guidelines and recommendations generally support PND screening in a range of healthcare settings, including primary care settings [4]. Nonetheless, in Australia, one in five women still do not receive perinatal mental health screening in line with current recommendations [5]. Encouragingly, a systematic review demonstrated that PND screening by a broad range of healthcare workers is acceptable to stakeholders [6], indicating that there is potential for expanded roles in this area to improve early detection. However, none of the studies included in this review focused on the acceptability of screening by one of the most accessible primary healthcare professionals, namely pharmacists. There is a need for further research exploring pharmacists’ roles in providing public health services [7].

Given pharmacists’ frontline roles and regular contact with consumers [8], it is not surprising that pharmacist-delivered screening for a range of illnesses has been shown to be acceptable to consumers and other healthcare professionals [9]. However, research exploring pharmacist-delivered screening for mental illness is minimal. Evaluations of pharmacist-delivered screening for mental illnesses, such as depression, have been exploratory in nature and demonstrate the feasibility of such services [10]. For example, a pilot study in Bulgaria showed that pharmacists were able to detect depression in chronically-ill patients, and indicated that depression screening in pharmacies should be encouraged [11]. Research exploring pharmacist-delivered PND screening, specifically, is limited, but pharmacists support families in many ways during the perinatal period through medication therapy management and other services (e.g., infant feeding support) [12]. There is evidence to suggest that pharmacists are willing to take on roles in perinatal mental healthcare [13], with measurement instruments exploring pharmacists’ knowledge and attitudes in this area having been developed [14, 15]. Recent evidence from interviews with perinatal women in Australia demonstrates that they are also generally accepting of pharmacists’ roles in this area if pharmacists had appropriate training [16]. Examining pharmacists’ perspectives of PND screening, specifically, is necessary to inform the development of such a service in an acceptable manner [6].

### Aim

This study aimed to explore pharmacists’ views of pharmacists’ roles in PND screening, as well as training and resource needs for PND screening in community pharmacy settings. The specific objectives were to explore pharmacists’ perspectives of (1) pharmacists’ roles in PND screening, with a focus on implications for acceptability and (2) training and resource needs for delivering PND screening in community pharmacies.

### Ethics approval

This study was approved by the Human Research Ethics Committee at The University of Sydney (Project Number: 2020/661).

## Method

### Study instrument

A questionnaire exploring PND screening acceptability and willingness comprising a section with 42-Likert scale response type (survey) questions, a demographics section and an open-ended response section was developed by academic members of a research team with expertise in pharmacy, business, psychiatry, and perinatal epidemiology, guided by published literature [6, 13, 15, 17] and the Theoretical Framework of the Acceptability (TFA) of healthcare interventions [18]. The Research Electronic Data Capture (REDCap) was used for data collection by storing and disseminating the questionnaire to pharmacists in Australia [19]. The focus of this manuscript is the analysis of the open-ended response section, only, and the demographics of participants who responded to these open-ended questions. The open-ended response section was composed of three questions to gain deeper insights on pharmacists’ perspectives and acceptance of pharmacists’ potential roles in PND screening, as well as training and resource needs to deliver such a service. The three questions were:

- ***Question one***: *Please share your thoughts on pharmacist’s roles in perinatal depression screening.*
- ***Question two***: *Please share your thoughts on what training would be needed for pharmacists to confidently conduct PND screening.*
- ***Question three***: *Please share your thoughts on what resources are needed for community pharmacy-based PND screening.*

### Participant recruitment and data collection

The questionnaire was advertised by national and state-based pharmacy organisations (e.g. through member communications), and promoted through social media platforms by members of the research team in Australia (November 2020–October 2021). Pharmacists that completed the survey were offered a $20 gift card for their participation.

### Data analysis

Data from REDCap were downloaded into Excel where it was analysed, guided by published qualitative analysis methods [18, 20–22]. Firstly, one author (CS) familiarised herself with the responses to each of the three questions, through multiple readings of the data, and recorded initial impressions of the data [20]. Individual responses were then condensed, which helped to identify the key meanings of the responses [20]. Paragraphs that conveyed different key meanings were accordingly separated into sentences [21]. Codes were created, which were composed of a few words capturing the main idea [20], whereby responses from each of the three open-ended questions underwent inductive coding, separately [20, 21]. Subsequently, codes were grouped into different categories based on their underlining meaning and connection to one another [20]. Moreover, for question one (pharmacists’ roles in PND screening), subcategories and categories were developed from the codes [20]. At each step of this process, CS consulted with two co-authors, SE and CRG, for discussion about the analyses.

Questions two (training requirements) and three (PND resources) were solely analysed by inductive content analysis (using the aforementioned process) as the responses provided specific details and suggestions relating to training and resources for PND screening, only. However, responses to question one regarding pharmacists’ roles allowed for further analyses through mapping of subcategories to the TFA. Subcategories generated from responses to question one were further analysed by mapping each subcategory to the TFA. The TFA is composed of seven constructs that influence acceptability, which are presented in Table 1. Guided by the methods employed in published literature [22], subcategories were mapped to one or more of the seven constructs of the TFA.

**Table 1:** Summary of Sekhon, Cartwright and Francis [18] theoretical framework of acceptability

Construct	Definition	Constructs adapted to community pharmacist-delivered PND screening
Affective attitude	“How an individual feels about taking part in an intervention” [18]	How pharmacists feel about community pharmacists taking part in PND screening
Burden	“The perceived amount of effort that is required to participate in the intervention” [18]	The perceived amount of effort that is required to participate in community pharmacist-delivered PND screening
Ethicality	“The extent to which the intervention has good fit with an individual’s value system” [18]	The extent to which community pharmacist-delivered PND screening has a good fit with the pharmacists’ value system
Intervention coherence	“The extent to which the participant understands the intervention and how it works” [18]	The extent to which pharmacists understand PND screening and how it works
Opportunity cost	“The extent to which benefits, profits, or values must be given up to engage in an intervention” [18]	The extent to which benefits, profits, or values must be given up to engage in community pharmacist-delivered PND screening
Perceived effectiveness	“The extent to which the intervention is perceived as likely to achieve its purpose” [18]	The extent to which community pharmacist-delivered PND screening is perceived as likely to achieve its purpose
Self-efficacy	“The participant’s confidence that they can perform the behaviour(s) required to participate in the intervention” [18]	Pharmacists’ confidence that they can perform the behaviour(s) required to participate in community pharmacist-delivered PND screening

*PND* Perinatal depression

Each stage of the content analysis of the three open-ended questions was led by CS who has experience in perinatal mental health education. This was done in collaboration with two co-authors and experienced researchers, (SE), a pharmacist academic and Mental Health First Aid Instructor, and CRG, a perinatal epidemiologist and academic. Using an iterative process, co-authors SE, CS and CRG met multiple times until consensus was reached on codes, categories and subcategories as well was the deductive mapping of the subcategories to the TFA constructs for question one.

In addition, a second, independent coder (LP) also familiarised themselves with 30% of the data and analysed this subsample of data. LP is a community, hospital, and research pharmacist with expertise in PND screening research. CS and LP compared their respective coding and TFA mapping and discussed any discrepancies until consensus was reached. As shown by literature, reflexivity on the position one has as a researcher when analysing and interpreting the findings of the research is crucial [23]. Hence, authors involved in data analysis met frequently during this phase of the research to reflect on and discuss the findings while considering their positions as researchers.

## Results

### Participant demographics

Out of 201 pharmacists who completed the questionnaire, 149 pharmacists responded to at least one of the three-open ended questions. Of the 149 participants that responded to question one, 75.8% identified as females, ages ranged between 21 and 66 years and 81.2% reported they were interested in further training (Table 2).

**Table 2:** Pharmacists’ characteristics and demographics

Characteristic	N (%)
Sample	N 149 (100)
*Age range (mean, SD)*	21–66 (33, 9)
*Gender*	
Female	113 (75.8)
Male	35 (23.5)
Prefer not to answer	1 (0.7)
Cultural background or ethnicity*	
Australian	100 (67.1)
Asian	56 (37.6)
Other	13 (8.7)
Prefer not to answer	6 (4)
Middle East	2 (1.3)
New Zealand	2 (1.3)
*Year of registration as pharmacist (range)*	1975–2022
1975–2000	15 (10.1)
2001–2022	134 (89.9)
Main area of practice*^#^	
Community pharmacy employee	105 (70.5)
Hospital pharmacy	23 (15.4)
Community pharmacy owner	21 (14.1)
Consultant pharmacy	20 (13.4)
Other	8 (5.4)
Pharmaceutical industry	6 (4)
Academia	5 (3.4)
Not currently working	3 (2)
*Defined area of practice*	
No	131 (87.9)
Yes	13 (8.7)
Not sure	5 (3.4)
*Have your personally ever experienced a mental health problem?*	
No	77 (51.7)
Yes	58 (38.9)
Prefer not to answer	14 (9.4)
*Has any member of your immediate family (mother, father, siblings) ever experienced a mental health problem?*	
Yes	84 (56.4)
No	53 (35.6)
Prefer not to answer	12 (8)
*Have you ever completed further training in mental health?*	
No	92 (61.7)
Yes	57 (38.3)
*Please estimate the proportion of your patients who are pregnant or postpartum (12 months post-delivery)?*	
None	4 (2.7)
Not sure	22 (14.8)
< 5%	53 (35.6)
5–25%	63 (42.3)
26–50%	4 (2.7)
51–75%	3 (2)
76–100%	0 (0)
*I would be interested in receiving further training to deliver screening and support services for women at risk of perinatal depression*	
Yes	121(81.2)
No	28 (18.8)

*Participants could select multiple options

^#^May include pharmacists with general or provisional registration

### Pharmacists’ roles in PND screening (question one)

Overall, participants felt that pharmacists have a role in PND screening, however, they also recognised potential challenges. Three overarching categories were identified from the analysis, which were: *PND screening in primary care settings will support the community*, *community pharmacy environment*, and *system and policy changes*. Ten subcategories resulted from these categories, which were mapped to the TFA constructs (Table 3).

**Table 3:** Summary of the analyses of the responses to question one (pharmacists’ roles)

Categories	Subcategories	TFA Constructs
PND screening in primary care settings will support the community	The need for PND screening in community pharmacies	Affective attitude
		Perceived effectiveness
	Customer -pharmacist relationship	Affective attitude
		Ethicality
	Accessibility	Affective attitude
		Perceived effectiveness
	Pharmacists are the link between detection and treatment	Intervention coherence
		Self-efficacy
Community pharmacy environment	Need for privacy	Affective attitude
		Burden
		Perceived effectiveness
	PND screening requires time	Burden
		Opportunity cost
System and policy changes	Pharmacist’s scope of practice	Self-efficacy
		Ethicality
		Intervention coherence
	Training	Self-efficacy
		Burden
		Intervention coherence
	Financial implications	Opportunity cost
		Burden
	Human resources	Burden
		Opportunity cost

*PND* Perinatal depression

#### Category 1: PND screening in primary care settings will support the community

This category, and associated four subcategories, illustrate pharmacists’ perspectives on the advantages of community pharmacist-delivered PND screening.

##### Subcategory 1.A: The need for PND screening in community pharmacies

This subcategory was mapped to the TFA constructs ‘affective attitude’ and ‘perceived effectiveness’. Most pharmacists expressed that PND screening in community pharmacies would be an important service. Participants reported that perinatal women often visit community pharmacies and, hence, community pharmacy would be an appropriate setting for PND screening:

*“Pharmacies are frequently visited by pregnant women and new mothers so it would make sense to provide PND screening in the pharmacy.” (ID84).*

Participants also reported that given pharmacists’ accessibility, screening in community settings may allow for greater reach of screening services:

*“… I think pharmacists would be able to reach a larger population and thereby identify those affected - this would be a valuable service to be able to provide to patients…” (ID199)*.

Participants also emphasised the benefits of offering PND screening services in community pharmacies in geographic areas which lack access to mental health services:

*“Our pharmacy is located rurally, and there is very limited mental health services and baby health services. Offering a service in screening for perinatal depression would have immense benefits for the community as it would be filling a gap that currently exists...” (ID5). *

##### Subcategory 1.B: Customer–pharmacist relationship

This subcategory was mapped to TFA constructs ‘affective attitude’ and ‘ethicality’. Participants described that they had multiple opportunities to engage with perinatal women given their existing relationships and frequent interaction with this population:

*“I see pregnant women daily and have built great relationship with them.” (ID17).*

This regular contact may be beneficial in allowing for opportunities for screening, with participants also highlighting that their relationships with consumers may improve comfort when disclosing mental health symptoms:

*“… Pharmacists may have a longer standing and more meaningful relationship with the patent *[patient]* that will help with honest answers to the screen.” (ID39).*

##### Subcategory 1.C: Accessibility

This subcategory was mapped to the TFA constructs ‘affective attitude’ and ‘perceived effectiveness’. Participants felt that community pharmacist-delivered PND screening service will provide an additional avenue for PND screening, due to high accessibility:

*“Pharmacists are the most accessible primary health professionals within a mother’s healthcare team to identify potential cases of perinatal depression. Therefore this practice should be encouraged.” (ID87).*

Moreover, participants felt that community pharmacies were an accessible setting for healthcare needs throughout the perinatal period:

*“I think it’s so important as we are the easiest access to a health professional for the community.  Mothers often come in for all the baby essentials which provides ample opportunity to assess the mental health of the parent.” (ID 196).*

##### Subcategory 1.D: Pharmacists are the link between detection and treatment

This subcategory was mapped to the TFA constructs ‘intervention coherence’ and ‘self-efficacy’. Participants felt that pharmacists could play important roles in detecting women at risk of PND and referring them to appropriate healthcare services, but recognised the need for further training to take on this role:

*“We can definitely be trained to be able to detect anyone suffering from a mental health issue, and make the necessary referral.” (ID59).*

Pharmacists also felt community pharmacy staff could encourage perinatal women to discuss and seek help for mental health symptoms:

*“A pharmacy assistant or pharmacist may be able to open a conversation to encourage women to discuss their mental health and screening and referral to appropriate services, doctor or psychologist...” (ID84). *

#### Category 2: Community pharmacy environment

The environment of the community pharmacy was an important factor thought to influence the extent of pharmacists’ potential contribution to PND screening and care.

##### Subcategory 2.A: Need for privacy

This subcategory was mapped to the TFA constructs ‘affective attitude’, ‘burden’ and ‘perceived effectiveness’. Pharmacists felt that PND screening could not be conducted over the counter:

*“It *[PND screening]* would have to be done in a clinic room not at thhe *[the]* counter.” (ID189).*

However, ensuring privacy was perceived as potentially burdensome, thereby posing a barrier to PND screening in community pharmacies:

*“I think it would be very valuable but challenging in some pharmacies due to business *[busyness]* or lack of private areas.” (ID147).*

##### Subcategory 2.B: PND screening requires time

This subcategory was mapped to the TFA constructs ‘burden’ and ‘opportunity cost’. Pharmacists described time constraints within pharmacies to be a major barrier to PND screening:

*“Pharmacists are the most easily accessible health professional but there is very limited time in a work day.” (ID179).*

Participants reflected on whether implementing PND screening services may limit pharmacists’ ability to provide care and services to other consumers:

*“I don’t think pharmacists should get involved in this area, as it takes away time from other patients…” (ID29).*

#### Category 3: System and policy changes

This category reflects the system and policy changes participants felt were necessary for delivery of PND screening in community pharmacies.

##### Subcategory 3.A: Pharmacists’ scope of practice

This subcategory was mapped to the TFA constructs ‘self-efficacy’, ‘ethicality’ and ‘intervention coherence’. There were mixed views regarding whether PND screening is within community pharmacists’ scope of practice. Some pharmacists believed that providing PND screening was not within their area of expertise and that PND screening was the responsibility of other healthcare professionals:

*“I do not believe that pharmacists are equipped or should be involved in screening for perinatal depression. This will be stepping into GP’s/obstetricians and psychologists boundaries.” (ID56).*

However, others reflected on the evolution of pharmacists’ roles in primary care, and the potential for pharmacists’ roles in screening:

*“I think screening for perinatal depression could form part of Pharmacist’s scope of practice and our expanding role in primary health care.” (ID78).*

##### Subcategory 3.B: Training

This subcategory was mapped to TFA constructs ‘self-efficacy’, ‘burden’ and ‘intervention coherence’. Pharmacists indicated that further training around PND was required:

*“I believe with the right training, pharmacist can provide benefit in perinatal depression screening.” (ID17).*

However, pharmacists felt that they would need to commit effort and time to upskilling in this area:

*“It may be hard to implement as it requires extensive training and confidence.” (ID58).*

##### Subcategory 3.C: Financial implications

This subcategory was mapped to the TFA constructs ‘opportunity cost’ and ‘burden’. Participants felt that remuneration was a prerequisite to delivering PND screening:

*“… remuneration would be essential to providing this service adequately.” (ID136).*

However, they also reflected on whether the financial investment would be worthwhile:

*“In terms of application to community pharmacy I am unsure if the condition of PND is common enough to warrant the cost of training/implementing a service.” (ID124).*

##### Subcategory 3.D: Human resources

This subcategory was mapped to the TFA constructs ‘opportunity cost’ and ‘burden’. To perform screening effectively, participants indicated that an adequate number of staff were needed and the lack of sufficient workforce in community pharmacy was seen as a potential barrier:

*“… I don’t believe it is possible to give it the time and attention it deserves if there is only 1 pharmacist on duty. You would need at least 2 pharmacists…” (ID39).*

Pharmacists also considered how the implementation of screening services would impact their workload and performance:

“[G]*reat in theory but in reality, its* [sic] *another increase in pharmacist work loads.* [S]*o either pharmacists work harder and put themselves at risk or they sacrifice some other part of their work.” (ID125).*

### Training requirements (question two)

This question investigated participants’ (n = 148) thoughts on pharmacists’ training needs - three categories emerged: *Training content, Training delivery* and *Training length*.

#### Category 1: Training content

Pharmacists acknowledged the importance of existing general mental health training, as well as the need for more specialised training in PND screening:

*“Mental Health First Aid is a good start but also guidelines of the correct questions to ask and the services available to help in referral and treatment.” (ID84).*

Pharmacists offered suggestions for the content of the training:

*“CPD* [continuing professional development] *-style courses on perinatal depression screening, covering issues such as the screening process, the factors taken into consideration and score interpretation. Also the pharmacy environment and other business issues.” (ID119).*

Pharmacists also recognised the need for extensive training on interpreting scores:

*“Workshop where we get a template and run through the scores and what to do with the scores.” (ID73).*

Another commonly identified element of training was communication:

*“Training on how to approach patients with sensitivity (eg types of questions and how to ask them)…” (ID131).*

Participants also stressed the importance of establishing appropriate referral pathways:

*“Referral options would also need to be discussed, as for community pharmacists I would imagine the appropriate referral would be either to the patient’s GP, midwife, private doctor or, if q10 is answered as yes (suicide risk) how we as pharmacists would arrange for the patient to be taken to the local ED* [emergency department] *for mental health assessment.” (ID172).*

#### Category 2: Training length

There were opposing views on the length of training, whereby some responses highlighted the need for in-depth, longer training:

*“An extensive training course would need to be provided.” (ID78).*

While others disagreed:

*“Training should be brief, if anything, enough just to identify perinatal depression in women so we can refer to a doctor or other health professional.” (ID57).*

#### Category 3: Training delivery

There were mixed views on how training should be delivered, with some preferring online delivery:

*“Online course should suffice.” (ID100)*.

And, others reporting a preference for in-person training:

*“So* [I] *believe pharmacist should be given the opportunity to do face to face training…” (ID52).*

However, some participants recommended a mix of both modalities:

*“*[O]*nline and f2f* [face to face] *training such as vaccine administration training.” (ID201).*

### PND resources (question three)

Data analysis of responses (n = 147) resulted in three categories: *Adapting community pharmacy operating structures*, *Pharmacist-specific resources* and *Consumer-specific resources*.

#### Category 1: Adapting community pharmacy operating structures

This category referred to resources that needed to change or be available to deliver PND screening:

*“A consultation room, training, remuneration for their time and easy to contact professionals where help is required” (ID7).*

Furthermore, there was a reported need for greater staff numbers:

*“… staffing requirements (at least 2 pharmacist present) so that 1 pharmacist could conduct screening” (ID50).*

Participants also highlighted the importance of being paid:

*“Remuneration!! As pharmacists we are … willing to provide primary care to ur* [your] *communities but we cannot work for free!” (ID146).*

#### Category 2: Pharmacist-specific resources

Pharmacists felt that there was a lack of information for them in this area and wanted readily-available pharmacist-specific resources:

*“Just any information, I haven’t really come across it unless I specifically look for as cpd* [continuing professional development] *activities to complete.” (ID185).*

Participants also provided suggestions on their preferences for resource content:

*“How to use the Edinburgh scale. How to interpret and what to do with women who test positive.” (ID 42).*

They also wanted resources that could direct them to other points of care:

*“… places to seek help eg. Mother’s groups, PANDA* [Perinatal Anxiety & Depression Australia].” *(ID36).*

#### Category 3: Consumer-specific resources

This category reflects pharmacists’ preferences for resources that they provide to consumers:

*“Informative leaflets - to give to patients regarding PND Resources - hotlines, websites, referral screening kit - including the questionnaire itself .” (ID103).*

Pharmacists highlighted the importance of developing user-friendly resources:

*“And* [An] *infographic that you could share with patients to help them understand their results. A booklet that explains screening process from beginning to end that pharmacists can refer to.” (ID147).*

## Discussion

This exploratory study demonstrates that pharmacists are generally accepting of pharmacists’ potential roles in PND screening, due to pharmacists’ accessibility and existing relationships with consumers. Nonetheless, factors such as staffing, privacy, time and remuneration were identified as integral for pharmacists’ roles in this area. Moreover, participants also raised concerns regarding their self-efficacy in terms of providing PND screening and emphasised the need for pharmacist-specific training focusing on PND screening, communication and referral pathways. Furthermore, operating structures and policies as well as developing pharmacist- and consumer-specific resources were considered essential for pharmacist-delivered PND screening.

Although participants recognised the potential benefits of PND screening in community pharmacies, they indicated the need for support from the wider healthcare system including collaboration with other healthcare professionals. Research on healthcare professionals’ perspectives on pharmacist-delivered screening is limited; however, available research demonstrates that generally other healthcare professionals are accepting of pharmacist-delivered screening for a range of medical conditions and risk factors [9]. Furthermore, perinatal women have also indicated that pharmacist-delivered PND screening and care could be beneficial [16]. There has been a shift to community-based primary care for many mental illnesses, resulting in many services and supports now being available through community pharmacies, including clozapine dispensing [24]. Further, pharmacists’ roles in mental healthcare are increasingly going beyond medication supply, with 85% of Australian pharmacists having interacted with a person at risk of suicide [25]. Nonetheless, future research exploring other healthcare professionals’ perspectives of pharmacist-delivered PND screening is warranted.

Study findings are supported by the broader literature pertaining to the accessibility of community pharmacists. In Australia, community pharmacies are highly accessible healthcare settings, and annualy, consumers visit pharmacies 18 times, on average [26]. The results of this study indicate that pharmacists’ accessibility may be especially important in geographic areas that lack access to mental health services. This aligns with another study which also demonstrated the importance of mental health screening and referral opportunities in rural pharmacies [27]. Nonetheless, participants indicated the need for pharmacist-specific training focusing on referral, PND screening, result interpretation and communication. Studies have, indeed, shown that knowledge around PND differs amongst healthcare providers, suggesting further PND education is warranted [28, 29]. Participants in the current study had varying views pertaining to the mode of delivery and length of training. Literature demonstrates that online training can be an effective method to improve healthcare professionals’ understanding around depression in the perinatal period [30, 31]. However, a scoping review demonstrated that there is no ideal or preferred training mode and length with regards to perinatal mental health training [32]; hence, further research exploring these factors is needed.

The findings of the current study highlighted the need for a consultation room to ensure privacy for the provision of pharmacist-delivered PND screening and care. Research exploring the provision of pharmacist-led mental health interventions often require participating pharmacies to have consultation rooms [33]. The lack of privacy in pharmacies is an important factor to address since it may prevent consumers from having confidential conversations with pharmacists [34]. Hence, the availability of a private space in community pharmacies is likely to be essential for the provision of pharmacist-delivered PND screening.

### Strengths and weaknesses

This exploratory study is among the first to investigate pharmacists’ roles in PND screening. However, volunteer bias is one potential limitation of this study since it was a self-administered questionnaire, and it is possible that those more interested in this topic were more likely to respond. While responses were collected anonymously which may have reduced the potential for bias, this also presents a potential limitation, in that a response rate could not be calculated. Moreover, most participants identified as females which could have potentially introduced bias; however, this is unlikely to be due to the nature of the topic (PND) and is likely a reflection of the pharmacy workforce in Australia which is 64% female [35]. This study adds to the evidence base regarding the acceptability of pharmacist-delivered screening and provides guidance for the development and implementation of future perinatal mental health training and services specific to community pharmacies.

## Conclusion

Participants were generally accepting of potential roles for pharmacists in PND screening, which may be facilitated by pharmacists’ accessibility and trusting relationships with consumers in community pharmacy settings. However, concerns around pharmacists’ scope of practice were raised and the development of pharmacist-specific resources and training was considered necessary. Findings may be used to guide the development of community pharmacist-specific PND training and resources, as well as the design, delivery and evaluation of a pharmacist-delivered PND screening service in community pharmacy settings. While this qualitative study provides valuable insights regarding pharmacists’ perspectives and acceptability on their role in PND screening future studies which use quantitative measure of acceptability to measure this construct among larger, representative samples of pharmacists are needed. Further research exploring other healthcare professionals’ views of pharmacist-delivered PND screening is warranted, and may help guide the establishment of referral pathways for this purpose.
